# European collaborations on medicine and vaccine procurement

**DOI:** 10.2471/BLT.21.285761

**Published:** 2021-08-20

**Authors:** Sabine Vogler, Manuel A Haasis, Rianne van den Ham, Tifenn Humbert, Sarah Garner, Fatima Suleman

**Affiliations:** a WHO Collaborating Centre for Pharmaceutical Pricing and Reimbursement Policies, Gesundheit Österreich GmbH, Stubenring 6, 1010 Vienna, Austria.; b WHO Collaborating Centre for Pharmaceutical Policy and Regulation, Utrecht University, Utrecht, Netherlands.; c World Health Organization Regional Office for Europe, Copenhagen, Denmark.; d WHO Collaborating Centre for Pharmaceutical Policy and Evidence Based Practice, University of KwaZulu-Natal, Durban, South Africa.

## Abstract

To ensure equitable access to medicines and vaccines, organizational efforts and purchase volumes have been pooled in joint procurements and negotiations for decades in some regions of the world, as well as globally through supranational procurement mechanisms. In Europe, countries started to collaborate on procurement and negotiations recently when it became increasingly difficult to ensure access to high-priced medicines, even in high-income countries. Two European country collaborations (the Nordic Pharmaceutical Forum and the Baltic Procurement Initiative) have successfully concluded at least one joint tender process for medicines and vaccines and the Beneluxa Initiative has concluded its first successful joint price negotiation. This article describes the experiences of these country collaborations. Challenges observed included: legal barriers; institutional and organizational differences between health-care systems in member countries; and the risk that suppliers will be reluctant to cooperate with country collaborations. Although these collaborations helped improve access to medicines and vaccines for the countries involved, in situations such as a global health crisis, larger-scale, more-inclusive initiatives are needed. In the current coronavirus disease 2019 (COVID-19) pandemic, COVID-19 Vaccines Global Access (COVAX) initiative established a global procurement mechanism to ensure the equitable distribution of COVID-19 vaccines globally. Despite differences in organization and scale, the European country collaborations and COVAX have some similarities: (i) their success depends on the increased purchasing power associated with pooled order volumes; (ii) expert knowledge and previous procurement experience is pooled; (iii) they perform other collaborative activities that go beyond procurement alone; and (iv) they actively involve external partners and stakeholders.

## Résumé

Depuis des décennies, certaines régions du monde ont uni leurs efforts pour s'organiser, négocier et effectuer des achats groupés de grandes quantités afin d'assurer un accès équitable aux médicaments et vaccins. Des mécanismes d'acquisition supranationaux ont fait de même à l'échelle planétaire. En Europe, des États ont récemment commencé à collaborer en matière d'achat et de négociation lorsqu'il est devenu de plus en plus difficile de garantir l'accès à des médicaments coûteux, y compris dans les pays à haut revenu. Deux collaborations entre pays européens (le Forum pharmaceutique nordique et l'Initiative d'acquisition de la Baltique) ont mené à bien au moins un processus d'offre conjoint pour des médicaments et vaccins, tandis que l'Initiative Beneluxa a conclu sa première négociation tarifaire conjointe. Cet article décrit les expériences liées à ces collaborations entre nations. Plusieurs défis se sont posés, notamment des obstacles juridiques; des différences institutionnelles et organisationnelles entre les systèmes de santé des États membres; et enfin, le risque que les fournisseurs soient peu enclins à accepter ces collaborations entre pays. Bien que ces collaborations aient amélioré l'accès aux médicaments et vaccins pour les pays impliqués, des initiatives plus globales et à plus grande échelle sont nécessaires dans des situations telles qu'une crise sanitaire mondiale. Durant l'actuelle pandémie de maladie à coronavirus 2019 (COVID-19), l'initiative COVAX (COVID-19 Vaccines Global Access) a abouti à un dispositif d'approvisionnement mondial pour veiller à distribuer équitablement des vaccins contre la COVID-19 dans le monde. Malgré des variations d'organisation et d'échelle, les collaborations entre États européens partagent des similitudes avec le COVAX: (i) le succès de ces deux démarches dépend d'un accroissement du pouvoir d'achat combiné à des volumes de commande groupés; (ii) elles mettent en commun les connaissances approfondies et expériences passées; (iii) elles mènent d'autres activités collectives qui dépassent le simple cadre de l'acquisition; et enfin, (iv) elles impliquent activement une série d'intervenants et de partenaires externes.

## Resumen

Para garantizar un acceso equitativo a los medicamentos y las vacunas, los esfuerzos organizativos y los volúmenes de compra se han unido en adquisiciones y negociaciones conjuntas durante décadas en algunas regiones del mundo, así como a nivel mundial a través de mecanismos de adquisición supranacionales. En Europa, los países empezaron a colaborar en las adquisiciones y negociaciones recientemente, cuando se hizo cada vez más difícil garantizar el acceso a los medicamentos con precios altos, incluso en los países de renta alta. Dos colaboraciones de países europeos (el Foro Farmacéutico Nórdico y la Iniciativa de Adquisición del Báltico) han concluido con éxito al menos un proceso de licitación conjunta de medicamentos y vacunas, y la Iniciativa Beneluxa ha concluido con éxito su primera negociación conjunta de precios. Este artículo describe las experiencias de estas colaboraciones entre países. Entre los retos observados se encuentran: las barreras legales, las diferencias institucionales y organizativas entre los sistemas sanitarios de los países miembros y el riesgo de que los proveedores se muestren reacios a cooperar con las colaboraciones entre países. Aunque estas colaboraciones ayudaron a mejorar el acceso a los medicamentos y las vacunas para los países implicados, en situaciones como una crisis sanitaria mundial, se necesitan iniciativas a mayor escala y más inclusivas. En la actual pandemia de la enfermedad por coronavirus (COVID-19), la iniciativa Acceso global a las vacunas de la COVID-19 (COVAX, por sus siglas en inglés) estableció un mecanismo de adquisición mundial para garantizar la distribución equitativa de las vacunas contra la COVID-19 en todo el mundo. A pesar de las diferencias de organización y escala, las colaboraciones de los países europeos y COVAX tienen algunas similitudes: i) su éxito depende del mayor poder adquisitivo asociado a los volúmenes de pedidos mancomunados; ii) se ponen en común los conocimientos de los expertos y la experiencia previa en materia de adquisiciones; iii) realizan otras actividades de colaboración que van más allá de la mera adquisición; e iv) implican activamente a socios y partes interesadas externas.

## ملخص

لضمان الحصول على الأدوية واللقاحات بصورة عادلة، تم تجميع الجهود التنظيمية وكميات الشراء في عمليات شراء ومفاوضات مشتركة لعدة عقود في بعض مناطق العالم، وكذلك على الصعيد العالمي من خلال آليات الشراء عبر عدة دول. بدأت الدول في أوروبا في التعاون فيما يتعلق بالتوريد والمفاوضات مؤخرًا، عندما أصبح من الصعب بشكل متزايد ضمان الحصول على الأدوية باهظة الثمن، حتى في البلدان ذات الدخل المرتفع. عقد كيانان للتعاون بين الدول الأوروبية (منتدى دول الشمال الأوروبي للصناعات الدوائية، ومبادرة البلطيق للتوريد) بنجاح عملية مناقصة مشتركة واحدة على الأقل للأدوية واللقاحات، واختتمت مبادرة بينيلوكسا (Beneluxa Initiative) أول مفاوضات مشتركة ناجحة بشأن الأسعار. تصف هذه المقالة تجارب هذه الكيانات التعاونية بين الدول. شملت التحديات التي تمت ملاحظتها ما يلي: الحواجز القانونية؛ الاختلافات المؤسسية والتنظيمية بين أنظمة الرعاية الصحية في الدول الأعضاء؛ ومخاطر إحجام الموردين عن التعاون مع الكيانات التعاونية بين الدول. على الرغم من أن هذه الكيانات التعاونية ساعدت في تحسين الحصول على الأدوية واللقاحات للدول المشاركة، في مواقف مثل الأزمة الصحية العالمية، هناك حاجة إلى مبادرات أوسع نطاقًا وأكثر شمولاً. في جائحة مرض فيروس كورونا 2019 (COVID-19) الحالية، أسست مبادرة الحصول العالمي على لقاحات (COVID-19 (COVAX آلية توريد عالمية لضمان التوزيع العادل للقاحات COVID-19 على مستوى العالم. على الرغم من الاختلافات في التنظيم والنطاق، إلا إن كيانات التعاون بين الدول الأوروبية وCOVAX بينها بعض أوجه التشابه: (1) يعتمد نجاحها على القوة الشرائية المتزايدة المرتبطة بأحجام الطلبات المجمعة؛ و(2) تجميع المعرفة المتمرسة والخبرة السابقة في مجال التوريد؛ و(3) تقوم بأنشطة تعاونية أخرى تتجاوز التوريدات وحدها؛ و(4) تشرك بنشاط شركاء وأصحاب مصلحة خارجيين.

## 摘要

为了确保公平获得药品和疫苗，几十年来在世界上的部分地区和全球范围内，某些国家会通过跨国采购机制汇集组织力量和采购单量以开展联合采购和谈判。由于即使在高收入国家，也越来越难以确保获得高价药品，因此，欧洲国家近期开始在采购和谈判方面达成联盟。两个欧洲国家联盟（北欧医药论坛和波罗的海采购联盟）成功地完成了至少一个药品和疫苗联合投标程序，比荷卢经济联盟也成功地完成了第一次联合价格谈判。本文描述了这些国家联盟的合作经历。观察到的挑战包括：法律障碍；成员国家保健体系之间的体制和组织差异；以及供应商不愿与国家联盟合作的风险。尽管这些联盟有助于改善相关国家获得药品和疫苗的情况，但在全球卫生危机等状况下，仍需采取更大规模、更具包容性的举措。在当前新型冠状病毒肺炎 (COVID-19) 大流行中，新冠疫苗全球供给计划 (COVAX) 建立了一个全球采购机制，以确保在全球范围内公平分配新冠疫苗。尽管在组织和规模上存在差异，但欧洲国家联盟与新冠疫苗全球供给计划还是存在一些相似之处：(i) 其成功取决于联合订单量所带来的购买力的提高；(ii) 汇集专业知识和以往的采购经验；(iii) 开展超越采购的其他合作活动；(iv) 积极地与外部合作伙伴和利益相关者进行交涉。

## Резюме

Чтобы обеспечить равный доступ к лекарственным средствам и вакцинам, на протяжении нескольких десятилетий усилия организаций и объемы закупок объединяются для проведения совместных закупок и переговоров в некоторых регионах мира, а также на глобальном уровне с использованием наднациональных механизмов закупок. Страны Европы начали сотрудничать в вопросах закупок и переговоров недавно, когда обеспечение доступа к дорогостоящим лекарственным средствам сильно затруднилось, даже в странах с высоким уровнем доходов. Две инициативы по сотрудничеству европейских стран (Фармацевтический форум стран Северной Европы и Инициатива по закупкам в странах Балтии) успешно завершили как минимум один совместный тендерный процесс для закупки лекарственных средств и вакцин, а Инициатива стран Бенилюкса завершила первые успешные совместные переговоры о ценах. В данной статье описывается опыт, полученный в ходе сотрудничества этих стран. Наблюдались следующие проблемы: юридические препятствия, институциональные и организационные различия между системами здравоохранения в странах-членах, а также риск того, что поставщики не захотят сотрудничать с объединениями стран. Несмотря на то что данное сотрудничество помогло улучшить доступ к лекарственным средствам и вакцинам в участвующих странах, ситуация глобального кризиса здравоохранения требует более масштабных и всеобъемлющих инициатив. В рамках текущей пандемии коронавирусной инфекции 2019 года (COVID-19) инициатива глобального доступа к вакцинам против COVID-19 (COVAX) создала глобальный механизм закупок для обеспечения справедливого распределения вакцин против COVID-19 во всем мире. Несмотря на различия в организации и масштабе закупок, сотрудничество европейских стран и COVAX имеют некоторые сходства: (i) успешность их деятельности зависит от увеличения покупательной способности, связанной с объемами объединенных заказов; (ii) объединение экспертных знаний и предыдущего опыта закупок; (iii) сотрудничество в других сферах, выходящих за рамки одних лишь закупок; (iv) активное привлечение внешних партнеров и заинтересованных сторон.

## Introduction

Access to essential medicines has become a major challenge globally. Even high-income countries with health-care systems based on social solidarity are increasingly struggling to afford the high price of medicines.^1^ In response, governments have been optimizing their policies on pricing and have piloted and implemented new policies.^2^ New initiatives include efforts to improve transparency on pricing and to strengthen collaboration, for example, through pooled procurement and joint price negotiations.^3^

Although marketing authorization has been harmonized among countries of the European Union, the pricing and reimbursement of medicines remain national competences. In Europe, national decision-making on pharmaceutical pricing and reimbursement is supported by sharing information and experience with pricing policy through, for example, the Pharmaceutical Pricing and Reimbursement Information network or the Piperska group,^4^^,^^5^ which are informal collaborations of public authorities and health-care funders that have been in place for more than a decade. Furthermore, European Union Member States and other stakeholders have cooperated on health technology assessment (mainly to improve methods), for instance, through the European Network for Health Technology Assessment (EUnetHTA), which is coordinated by the European Commission.^6^

However, compared with other regions of the world, Europe has had limited experience with cross-country collaborations on procurement. In contrast, for decades countries in the Americas have been using the Pan American Health Organization’s (PAHO) Revolving Fund for vaccines, syringes and related supplies and PAHO’s Strategic Fund for other essential medicines.^7^^,^^8^ Moreover, since the late 1970s, Persian Gulf states have collaborated through the Gulf Cooperation Council to procure medicines jointly.^8^^,^^9^ In some regions, government-led procurement initiatives are supplemented by global procurement mechanisms for medicines and vaccines coordinated by supranational institutions. Some examples are the Global Drug Facility for tuberculosis drugs, the supply division of the United Nations International Children's Emergency Fund (UNICEF) and the recently established procurement mechanism by COVID-19 Vaccines Global Access (COVAX) initiative for coronavirus disease 2019 (COVID-19) vaccines.^10^^,^^11^ Whether country-led or coordinated by supranational institutions, pooled procurement has provided benefits; for example: (i) lower purchase prices and thus improved access to medicines and vaccines; (ii) reduced operational costs and administrative burden; (iii) improved quality assurance; (iv) better governance; and (v) greater equity.^12^^,^^13^

In 2014, the European Commission approved the Joint Procurement Agreement for medical countermeasures,^14^ which established a joint procurement procedure for items such as vaccines, antivirals and other emergency supplies. However, the agreement was not intended to be used to procure high-priced medicines, such as orphan drugs for rare conditions and oncology medicines.^15^

In recent years, two main strategies for ensuring access to medicines have become prominent in Europe. First, procurement in general has risen up the political agenda and is increasingly considered an important topic, as confirmed by the European Union’s adoption of a pharmaceutical strategy for Europe.^16^ The strategic relevance of procurement was also highlighted by discussions on strategic procurement initiated by the World Health Organization’s (WHO) Regional Office for Europe in 2016.^17^^,^^18^ Second, some countries of a similar size and income level have joined forces to establish so-called voluntary collaborations.^19^ These collaborations are coordinated by national governments that wish to increase the affordability of medicines by aligning their national pricing and procurement procedures – they do not involve regional or supranational organizations.

Examples of such voluntary collaborations are the Baltic Procurement Initiative (involving Estonia, Latvia and Lithuania), the Beneluxa Initiative on Pharmaceutical Policy (involving Austria, Belgium, Ireland, Luxembourg and the Netherlands), the Fair and Affordable Pricing initiative (involving mainly countries in Central and Eastern Europe), the Nordic Pharmaceutical Forum (involving Denmark, Iceland, Norway and Sweden, with Finland as an observer) and the Valletta Declaration (involving mainly Mediterranean countries).^20^ Apart from the 2012 Baltic Procurement Initiative, all were founded in 2015 or later. Most of these collaborations aim to cooperate in areas such as information sharing, horizon scanning (i.e. identifying potential future medicines in the research and development pipeline) and health technology assessment. The Beneluxa Initiative does not perform pooled procurement but does conduct joint pricing negotiations;^21^ initially these were for orphan medicines but now include medicines with a large budgetary impact. 

Three collaborations (i.e. the Baltic Procurement Initiative, the Beneluxa Initiative and the Nordic Pharmaceutical Forum) have successfully concluded at least one joint cross-country tender process or price negotiation. The aim of this paper was to describe the experience of these three European country collaborations as the lessons learnt may be of interest to other countries that wish to carry out joint procurement or price negotiations and may be applicable to procurement mechanisms coordinated by supranational organizations. Their experience may also be relevant for WHO’s European Programme of Work, which designates pooled procurement initiatives as a key priority.^22^ In addition, we compare the experiences of these three collaborations with those of the new global COVAX mechanism for procuring and distributing COVID-19 vaccines.

## European experience

The three European country collaborations we studied conducted either a joint procurement process or joint price negotiations, in addition to other activities. The Beneluxa Initiative adopted a value-chain perspective, which involved monitoring medicines during: (i) pre-launch research and development; (ii) the peri-launch period after marketing authorization and before launch onto the market (when pricing and reimbursement decisions are made); and (iii) the post-launch period, when measures to promote appropriate prescribing, dispensing and use of medicines can be implemented.^23^

In addition to joint price negotiations, the Beneluxa Initiative collaborated on horizon scanning and health technology assessment, which resulted in the alignment of timelines and health technology assessment methodologies within the collaboration. Further, a joint template was prepared to help manufacturers submit dossiers for joint assessments.^24^^,^^25^ Countries in the Nordic Pharmaceutical Forum also collaborated on horizon scanning, and members of the Baltic Procurement Initiative established a lending agreement to help each other when there were shortages by lending medicines or medical devices without charge. However, the following discussion focuses on joint procurement and joint price negotiations. The main characteristics of, and the key lessons learnt in, these three collaborations are summarized in Box 1.

Box 1 **Characteristics of, and lessons learnt from, three European country collaborations on procuring medicines and vaccines, 2010–2021**

**Nordic Pharmaceutical Forum**

Established: 2015

Countries involved: Denmark, Iceland, Norway and Sweden

Activity studied: Joint procurement of medicines

Activity history:

- One successful joint procurement (concluded 2020)
- Second tender call in preparation (2021)

Key lessons learnt:

- Legal barriers can prevent participation
- Countries having similarly organized health and pharmaceutical systems is helpful
- Having a small market in a collaboration can be a drawback
- Substantial resources are required (minimum: one full-time member of staff)
- Extensive dialogue with potential suppliers in advance is necessary and beneficial
- Efficient and timely planning is needed
- Regular coordination meetings between countries are needed
- Logistics must be considered

**Baltic Procurement Initiative**

Established: Agreement 2012 (task force 2010)

Countries involved: Estonia, Latvia and Lithuania

Activity studied: Joint procurement of vaccines

Activity history:

- One failure (2015)
- Three successful joint procurements (2017–2018)
- First strategic procurement plan (2019)
- Preparation for the next joint procurement (2020)

Key lessons learnt:

- Collaboration becomes easier with experience (the beginning can be very challenging)
- Clarity is needed on legislation, regulations and procedures
- Simple procurement procedures are needed, including simple and short procurement documents
- Writing documents in English instead of national languages can be beneficial
- Rotating the lead procurement partner can be beneficial
- The work of technical experts can benefit from the interest and support of high-level policy-makers
- Voluntary participation of countries allows flexibility
- Increasing the number of participants can slow the procurement process
- A market feasibility analysis is important

**Beneluxa Initiative**

Established: 2015

Countries involved: Austria, Belgium, Ireland, Luxembourg and the Netherlands

Activity studied: Joint negotiations on medicines

Activity history:

- One joint negotiation failed (2017)
- One successful joint negotiation (2018)

Key lessons learnt:

- Failures can and will occur, but can enable further lessons to be learnt
- A value-chain approach requires collaboration on horizon scanning, health technology assessment and negotiations
- Joint negotiations can result in country-specific outcomes (e.g. different prices or reimbursement conditions)
- Legal barriers can present challenges
- Differences between countries in pricing and reimbursement processes can present challenges
- Negotiations can take time
- Successful negotiation depends on the value all parties (i.e. countries and companies) see in the agreement
- Basing negotiations on the added value of a medicine rather than on its price can encourage more countries to participate

Note: Information was obtained during interviews conducted between July and November 2018 with government officials involved in collaborations and from follow-up correspondence that took place in October 2019 and in January and June 2021.

### Nordic Pharmaceutical Forum

On 1 February 2020, the Nordic Pharmaceutical Forum entered into its first joint tender agreement after the successful conclusion of a technical procurement process lasting over a year. Preparations for the tender call dated back to the summer of 2017 when the Forum explored options for a pilot joint procurement exercise. In September 2018, the collaboration conducted a market survey to collect potential suppliers’ views on a preliminary list of suitable products. The first joint tender process focused on ensuring the availability of older medicines whose patent had expired (e.g. antibiotics such as ciprofloxacin, ceftriaxone and metronidazole) and for which it was difficult to find suppliers for Nordic countries because of their relatively small markets.

Details of the procurement were based on an extensive dialogue with potential suppliers. Before tenders were invited, 6 weeks of hearings were held with potential bidders and their comments were considered during preparation of the tender call documents. Denmark was the lead country and the tender call was based on Danish legislation. Norway and Iceland participated in this first Nordic tender process, whereas Sweden (which has a fragmented hospital sector) did not join in. There were several challenges, particularly for Iceland, because potential suppliers did not consider its market to be sufficiently attractive to apply for marketing authorization. Consequently, the Nordic Pharmaceutical Forum let the suppliers decide whether to include Iceland in their offers, with the result that Iceland received an offer from only one company. Overall, a sufficient number of bids was received for most tender calls to ensure adequate competition. In total, nine contracts were signed for medicines such as ondansetron, gentamicin, paracetamol, meropenem, anagrelide and methotrexate.^26^^,^^27^ Members of the Nordic Pharmaceutical Forum were reportedly satisfied with this first tender process. However, major challenges had to be addressed: (i) legal barriers had to be overcome; (ii) sufficient resources were required; and (iii) adequate time for planning was needed (Box 1).

In June 2021, the Nordic Pharmaceutical Forum issued another joint tender call, which this time included environmental criteria such as: (i) following good practice to ensure zero carbon emissions and clean wastewater at the bidder’s and their subsuppliers’ production sites; and (ii) making an effort to reduce greenhouse gas emissions from transport.^28^ Reportedly, Iceland has started to change its legislation to enable participation in future Nordic tenders.^20^

### Baltic Procurement Initiative

To date, the Baltic Procurement Initiative has focused on procuring vaccines and has successfully concluded three tender processes. In 2019, the collaboration exited the pilot phase and progressed towards a more strategic collaboration by developing the Baltic countries’ first joint procurement plan. However, the first joint tender call in 2015 was unsuccessful because no bids were received. Since then, the collaboration has paid greater attention to market analysis and increased its consultations with stakeholders before inviting tenders.

For each joint procurement process, a lead country is identified and other countries legally consent to it procuring products on their behalf. The lead country’s legislation is applied. For the first tender process, documents were prepared simultaneously in the national languages of the two participating countries. From the second joint procurement process onwards, all documents (with a few exceptions for legal reasons) were prepared solely in English, a foreign language in all Baltic countries. The Initiative aims to keep the procurement procedure as simple as possible and is committed to preparing simple, short documents. For each procurement, the three collaborating countries decide which will participate and which will be the lead country. Participation is voluntary and usually involves only those countries that have the particular vaccine in their immunization schedules.^20^

### Beneluxa Initiative

Using the value-chain approach, members of the Beneluxa Initiative on Pharmaceutical Policy first identify medicines of interest for collaborative action through horizon scanning, then they perform a joint health technology assessment and, finally, they conduct joint price negotiations. Collaborating countries do not all have to be involved in all steps of the process. Moreover, a successful joint negotiation does not necessarily mean that prices and reimbursement conditions are the same for all countries as the final decisions on pricing and reimbursement are taken nationally.

In July 2018, two countries (Belgium and the Netherlands) were involved in the first successful joint negotiations (for nusinersen, a medicine for spinal muscular atrophy) – a joint health technology assessment had been carried out to inform the negotiations. Reimbursement conditions differed slightly between the two countries and the negotiated price (i.e. the price agreed by payers but kept confidential) may also have differed. The Beneluxa Initiative aims to involve additional member countries in future negotiations.^29^

The Initiative has had limited experience with joint negotiations. To date, there have been only two: (i) the nusinersen negotiations; and (ii) earlier, unsuccessful negotiations on the combination of lumacaftor and ivacaftor, which are used for treatment of cystic fibrosis. The Beneluxa Initiative reported that motivating the pharmaceutical industry was difficult, particularly large multinational companies, to enter into negotiations with the collaboration. Additional problems were differences between countries in national pricing and reimbursement processes. Moreover, as Beneluxa joint procedures do not overrule national legislation and national procedures remain in force,^30^ member countries must come into legislative and procedural alignment with each other.

## Discussion

### European collaborations

In general, the procurement process for medicines and vaccines is prone to failure. Indeed, two of the three collaborations we analysed had unsuccessful negotiations and tender calls. There is a risk that suppliers may have limited interest in tender calls, or may lack the ability to respond to them, and that winning bidders may not meet their contractual obligations. These risks can be mitigated by having in-depth knowledge of the market (e.g. through consultation and dialogue with possible suppliers and by market research) but they can never be eliminated. Good planning is key for procurers but is resource-intensive. 

All three European collaborations were confronted with challenges resulting from legal, institutional and organizational differences between health-care systems in member countries and sometimes a change in legislation was required. For instance, Belgium had to alter its legislation to permit dossiers submitted by manufacturers to be written in English, which is the working language of the Beneluxa Initiative.^20^ The Nordic Pharmaceutical Forum and the Baltic Procurement Initiative opted to designate a lead country whose national legislation would be applied during joint procurements. In general, extensive efforts were required to align national procedures with the newly established processes of the country collaboration, especially in the starting phase.

All three collaborations followed the principle of voluntary participation, which meant that, for each collaborative action, member countries could choose whether or not to be involved. If a country decided to join an activity, it had to take on specific responsibilities.

### COVAX procurement mechanism

At the time of writing in 2021, the world is fighting one of the most serious health crises in history. Research and development into vaccines against severe acute respiratory syndrome coronavirus 2 (SARS-CoV-2) that are effective against viral mutations of concern is essential and global administration of these vaccines is urgently needed. Ensuring their supply to all eligible individuals requires actions that go far beyond what can currently be achieved by the government-led country collaborations described here.

In Europe, the European Commission has been coordinating the pooled procurement of COVID-19 vaccines for all European Union Member States because some states have relatively small markets or relatively low national income levels. This pooled effort, which is supported by investments such as advance purchase commitments,^31^ was able to secure vaccines for the European population.^32^

The COVID-19 pandemic highlights the vital need for further large-scale collaborations at the global level. In April 2020, the Access to COVID-19 Tools (ACT) Accelerator partnership was launched by the European Commission, WHO and other global health organizations with the aims of: (i) accelerating the development of vaccines, diagnostic tests and treatments for COVID-19; (ii) ensuring equitable global access to vaccines, tests and treatment; and (iii) strengthening health systems.^33^ Its vaccine pillar is COVAX, which is led by Gavi, the Vaccine Alliance, WHO and the Coalition for Epidemic Preparedness Innovations foundation. The COVAX Facility, which is the global procurement mechanism of the partnership, makes investments across the portfolio of promising vaccine candidates and negotiates contracts using the pooled purchasing power of participating countries. Through COVAX Facility mechanisms, vaccines will be distributed equitably as soon as they are available, for example, by richer countries donating excess doses.^34^ In addition, UNICEF, the world’s largest single vaccine buyer, has partnered with Gavi in the COVAX Facility to manage the procurement of COVID-19 vaccine doses, as well as their transport, distribution and storage. This partnership has led to the procurement and delivery of vaccine to 92 low-income and lower-middle-income countries and has supported procurement for 97 upper-middle-income and high-income countries.^35^

In terms of the number of countries and stakeholders involved and its institutional set-up, COVAX is not strictly comparable with cross-country, joint, procurement and negotiation collaborations involving only a few countries. Instead, COVAX enables countries without well-developed health systems to streamline their processes and provides technical support. In addition, it is a risk-pooling mechanism: participating countries do not know in advance which vaccines will eventually be successful. Importantly, COVAX member countries are not involved in negotiations or in the procurement process, as they are in the European collaborations we studied.

Nonetheless, country-led collaborations and COVAX have some common characteristics. First, all joint procurements benefit from the stronger purchasing power associated with pooled order volumes, which helps address problems with access to medicines. Second, joint procurement and negotiations can take advantage of existing knowledge and the experience gained during previous procurement processes: this is reflected by the prominent role played by expert organizations in procurement initiatives (e.g. by central procurement agencies in Denmark and Norway in the Nordic Pharmaceutical Forum and by UNICEF, which partnered with the PAHO Revolving Fund in some cases, in COVAX). Third, procurement and price negotiations constitute only one of several instruments in an end-to-end approach to improving access that is influenced by other, equally relevant policies. For example, the COVAX Facility is embedded in the ACT Accelerator, which additionally aims to encourage the development of effective vaccines, tests and treatments. Investment incentives, therefore, also play a major role. In country collaborations, joint procurement processes and negotiations form part of a larger portfolio of collaborative action, as reflected by the pharmaceutical value-chain approach chosen by the Beneluxa Initiative. Lastly, success depends on interactions with stakeholders (e.g. developers and suppliers), thereby achieving a beneficial situation for all. 

## Conclusions

Country collaboration on procurement is important for improving access to medicines and vaccines. In certain circumstances, as exemplified by the high global demand for vaccines and the need for their equitable worldwide distribution during the COVID-19 crisis, there appears to be no alternative to global procurement organized by a coalition of governments and supranational organizations. Further large-scale global procurement and price negotiations could help advance public health goals, particularly in low- and middle-income countries. However, it is unclear whether governments would be interested in extending mechanisms such as COVAX to other medicines and vaccines.

Regardless of whether or not countries collaborate, good practice is essential for public procurement. It is important to be prepared by achieving a good understanding of the needs of users and of the characteristics of products and the market. In addition, joint procurement processes face the challenges of legal barriers and of differences in organizational and financial structures between countries. In government-led collaborations, self-organization is also needed: a working structure must be established first and sufficient resources must be made available for the technical experts involved.

Given the need for up-front and continued investment in collaborations, it is important that outcomes are regularly monitored and evaluated so that operating structures and future investment can be adjusted if defined objectives are not fully met. The examples we studied show that collaborations on procurement and negotiations can produce positive results, although the time needed between the original conception and the establishment of a working structure may be substantial. Although further lessons can be expected from these European collaborations, today they can already serve as models for similar collaborations in other regions of the world.
